# Creating Inclusive Advance Care Planning Tools

**DOI:** 10.1093/geroni/igaa057.2720

**Published:** 2020-12-16

**Authors:** Robert Beringer, Gloria Gutman, Brian de Vries, Helen Kwan, Katrina Jang, Avantika Vashisht, Taranjot Kaur

**Affiliations:** 1 University of Victoria, Victoria, British Columbia, Canada; 2 Simon Fraser University, Vancouver, British Columbia, Canada; 3 San Francisco State University, San Francisco, California, United States; 4 Gerontology Research Centre, Vancouver, British Columbia, Canada; 5 Simon Fraser University Gerontology Research Centre, Vancouver, British Columbia, Canada

## Abstract

The cultural appropriateness of two ACP tools, My Wishes, My Care, and Conversation Starter Kit, are examined. In development by the BC Centre for Palliative Care, My Wishes, My Care was reviewed with: LGBT Non-Metropolitan Older Adults (6 focus groups, N=32); English/Chinese Speaking Older Adults (1 focus group, N=9); and English/Punjabi or Hindi Speaking South Asian Older Adults (1 focus group, N=5). The Conversation Starter Kit, developed by Ariadne Labs, was reviewed with focus groups of people whose loved one was/is in a Care Home: one LGBT group (N = 5); one South Asian group (N = 5). General feedback on both tools included: concerns over level of understanding required to use the tool; the role of family in ACP planning; cultural inclusivity (e.g., images and text) and design elements. The feedback will inform revision of these tools and assist organizations in developing inclusive ACP planning guides or educational materials.

